# First reported case of *Campylobacter lanienae* enteritis in a human

**DOI:** 10.1099/jmmcr.0.005045

**Published:** 2016-06-28

**Authors:** Simon Lévesque, Frédéric Lemay, Sadjia Bekal, Eric H. Frost, Sophie Michaud

**Affiliations:** ^1^​ Laboratoire de santé publique du Québec/Institut national de santé publique du Québec, Sainte-Anne-de-Bellevue, Québec, Canada; ^2^​ Faculté de Médecine et des sciences de la santé de l’Université de Sherbrooke, Sherbrooke, Québec, Canada; ^3^​ Centre de recherche du Centre hospitalier universitaire de Sherbrooke(CHUS), Sherbrooke, Québec, Canada

**Keywords:** *Campylobacter*, enteritis, campylobacteriosis

## Abstract

**Introduction:**

Campylobacters are the most frequently identified bacteria causing diarrhoea in humans worldwide. *Campylobacter lanienae* was isolated for the first time in 2000 from faecal samples of two asymptomatic abattoir workers in Switzerland during a routine hygiene screen, but has never been associated with human disease.

**Case presentation:**

At hospital admission, the patient reported diarrhoea, lower abdominal cramps, nausea, one episode of bilious vomiting and low-grade fever of 38 °C. The patient was having 10 or more diarrheic stools per day as well as during the night, and had noticed blood mixed with the stools on several occasions. Stool cultures were negative for species of *Salmonella* and *Shigella*, *Escherichia coli* O157:H7 and *Yersinia enterocolitica*, but were positive for *C. lanienae*. Identification was made by classical biochemical testing, as well as 16S rRNA gene and *cpn60* sequencing. The patient slowly improved without antibiotic treatment and was discharged nine days after admission with complete resolution of symptoms.

**Conclusion:**

On the whole it seems very likely that *C. lanienae* was the causative agent. Clinical microbiologists should be aware of this micro-organism which can be identified by phenotypic and molecular methods. The real burden of *C. lanienae* infection in humans might be underestimated and should be further investigated as a potential cause of human diarrhoea disease.

## Introduction

Campylobacters are the most frequently identified bacteria causing diarrhoea in humans worldwide (Allos, 2001). *Camplylobacter lanienae* was isolated for the first time in 2000 from faecal samples of two asymptomatic abattoir workers in Switzerland during a routine hygiene screen (Logan *et al.*, 2000), but has never been reported in humans since. *C. lanienae* has also been isolated from pig and wild boars faeces in Japan (Sasaki *et al.*, 2003, 2013), Hungary (Schweitzer *et al.*, 2011), Korea (Shin & Lee, 2009) and the USA (Jay-Russell *et al.*, 2012), from wild ruminants in Spain (Carbonero *et al.*, 2014; Navarro-Gonzalez *et al.*, 2014), from sheep in Turkey (Acik *et al.*, 2013) and Spain (Oporto & Hurtado, 2011), and from bovine faeces in Alberta (Inglis *et al*., 2003, 2004, 2005) as well as in Quebec (Guevremont *et al.*, 2008). Recently, the bacterium was also isolated from laboratory chinchillas in the USA (Turowski *et al.*, 2014). In this study we report what seems to be, to our knowledge, the first case of a symptomatic human infection with *C. lanienae*.

## Case report

In March 2009, a 39-year-old female sought treatment at the emergency department of the Centre Hospitalier Universitaire de Sherbrooke, reporting a 5-day history of diarrhoea, lower abdominal cramps, nausea, one episode of bilious vomiting and low-grade fever of 38 °C. She was having 10 or more diarrheic stools per day as well as during the night, and had noticed blood mixed with the stools on several occasions. She denied any infectious contact, recent antibiotic treatment or travel outside the province of Quebec. She was living on a hog farm and usually drank water from a private well. One year prior to the consultation, the patient had a diagnosis of irritable bowel syndrome and a colonoscopy performed then was normal. For the past few months, she was experiencing three loose stools a day. The patient had also been diagnosed with gastroesophageal reflux disease, hypothyroidism and refractory interstitial cystitis, and had a total hysterectomy. Her daily medication included esomeprazole, levothyroxine, phenazopyridine, amitriptyline, pentosan polysulfate sodium and pregabalin. Physical examination showed sinus tachycardia with normal blood pressure and a temperature of 37.5 °C. The abdomen was diffusely tender but soft with normal bowel sounds. The complete blood count, serum electrolytes, creatinine, and liver and pancreatic enzymes were normal. A pelvic and abdominal ultrasound showed only a left ovarian cyst. The patient was admitted to our hospital for intravenous hydration and observation. Because of the persistence of symptoms five days after admission, a colonoscopy was performed and revealed mild inflammation in the left colon. Biopsies confirmed discrete acute focal colitis. The *Clostridium difficile* toxin assay and a stool examination for ova and parasites were negative. Blood cultures were also negative. Stool cultures done two days apart were negative for species of *Salmonella* and *Shigella*, *Escherichia coli* O157:H7 and *Yersinia enterocolitica*, but showed a ‘*Campylobacter*-like’ organism. Because the patient’s symptoms were not compatible with a viral infection, no viral detection tests were performed. The patient slowly improved without antibiotic treatment and was discharged nine days after admission with complete resolution of her symptoms.

## Investigations

The stool sample was initially streaked on Karmali agar and incubated under microaerobic conditions generated in a jar with a CampyGen sachet (Oxoid) at 42 °C for 48 h. Small, grey, flat, irregular or round and moist colonies were observed but growth was poor compared to the *Campylobacter jejuni* control strain. The Gram stain revealed slender, curved Gram-negative rods. The organism was oxidase- and catalase-positive, hippurate-negative, indoxyl acetate-negative, resistant to both nalidixic acid and cephalothin, and susceptible both to erythromycin and ciprofloxacin by the disc diffusion method.

**Fig. 1.**
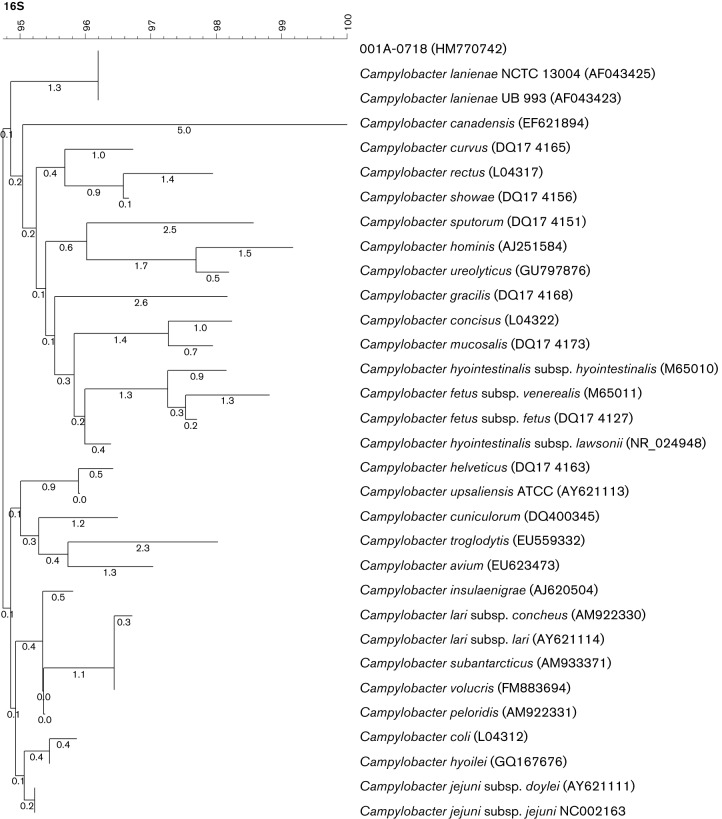
Phylogenetic tree based on the neighbour-joining method representing the genetic relatedness between partial 16S rRNA gene sequences for campylobacters. The bar represents the percentage of similarity, and numbers on the tree indicate internal branch distances within the resulting trees obtained by bootstrap analysis (1000 replicates). GenBank accession numbers are in parentheses.

The Laboratoire de Santé Publique du Québec (LSPQ) further identified the isolate by sequencing the *cpn60* gene (Hill *et al.*, 2006) and the 16S rRNA gene (Bekal *et al.*, 2006). BLAST analysis of the partial 16S rRNA gene sequence obtained (1221 bp), using the taxonomy browser of the National Center for Biotechnology Information (www.ncbi.nlm.nih.gov), revealed between 98 % and 100 % sequence identity with available *C. lanienae* sequences. The sequence was deposited in the GenBank database under accession number HM770742. Fig. 1 shows the phylogenetic position of the patient’s isolate (001A-0718) in a neighbour-joining tree representing the genetic relatedness between DNA sequences of the partial 16S rRNA gene of *Campylobacter* lineage. The isolate represented a distinct monophyletic lineage within the genus *Campylobacter* and was related to human strains NCTC 13004 and UB 993, the only two strains of *C. lanienae* previously isolated from humans (Logan *et al.*, 2000). Sequence comparison of our isolate with NCTC 13004 showed 99.43 % similarity for 1220 nucleotides. The partial sequence of the *cpn60* gene was also compared with cpnBD (Hill *et al.*, 2004) and the isolate was identified as *C. lanienae*.

**Fig. 2.**
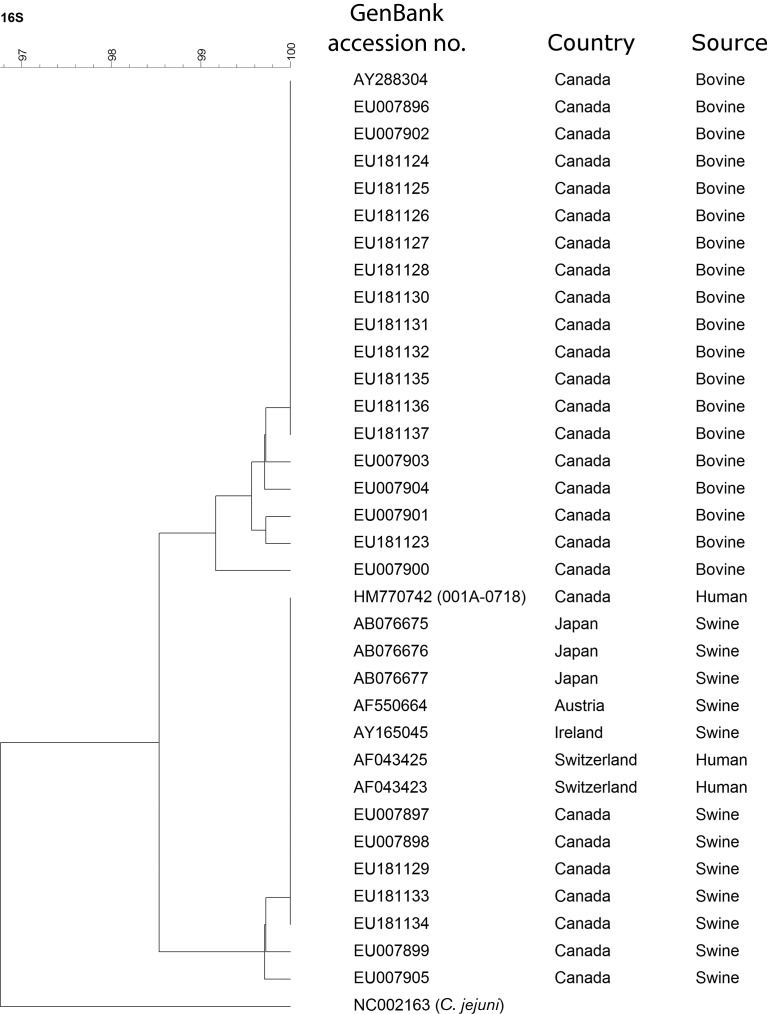
Phylogenetic tree based on the unweighted pair group method with arithmetic mean (UPGMA) method representing the genetic relatedness between sequences of the partial 16S rRNA gene for *C. lanienae*. The bar represents the percentage of similarity. *C. jejuni* was used as an outgroup.

Fig. 2 shows the phylogenetic position of our isolate in an unweighted pair group method with arithmetic mean (UPGMA) tree representing the genetic relatedness between DNA sequences of the partial 16S rRNA gene from *C. lanienae* isolated worldwide from human, bovine and swine faeces. Our isolate clustered with *C. lanienae* previously isolated from swine in Japan, Austria, Ireland, Switzerland and Canada, and the sequence analysed showed the same five nucleotide differences in regard to bovine isolates, as previously described by Guevremont *et al.* (2008). The fact that our isolate was resistant to cephalothin also supported the relationship with swine isolates, as bovine strains are typically susceptible to cephalothin (Shin & Lee, 2009).

## Discussion

In a PCR-based survey of *Campylobacter* associated with faeces collected from beef cattle, *Campylobacter* DNA was detected in 83 % of the samples (Inglis *et al.*, 2003). The most frequently detected species was *C. lanienae* (49 %), followed by *C. jejuni* (38 %), *Cmpylobacter hyointestinalis* (8 %) and *Campylobacter coli* (0.5 %). In another study, some cattle were found to be shedding exceptionally large numbers of cells (approximately 10^8^ cells g^−1^), and numbers of *C. lanienae* released in the environment in cattle faeces could exceed 10^12^ cells per animal per day(Inglis *et al.*, 2004). Inglis *et al*. reported that *C. lanienae* dwells in the large intestine, including the caecum, proximal ascending colon, distal descending colon, and rectum of cattle (Inglis *et al.*, 2005) and suggested that *C. lanienae* may be pathogenic to cattle (Inglis *et al.*, 2004).

Inglis *et al*. also observed that PCR-based detection methods were substantially more effective for detecting *C. lanienae* from faecal specimens than conventional culture methods (Inglis *et al.*, 2003). They isolated *C. lanienae* from cattle faeces, but found that cattle strains of the bacterium were very fastidious and did not grow on many of the commercial media commonly used to isolate campylobacters (Inglis & Kalischuk, 2003). The first isolation of *C. lanienae* was obtained on Campylosel agar, consisting of 5 % (v/v) blood in a Columbia agar base with the selective antibiotics cefoperazone, vancomycin and amphothericin B (Logan *et al.*, 2000). When comparing different culture media for growing *C. lanienae* from cattle faeces, Inglis and Kalischuk were only able to recover isolates from Karmali medium at 40 °C (Inglis & Kalischuk, 2003). *C. lanienae* is susceptible to polymyxin B (Logan *et al.*, 2000), which might explain why the organism cannot be recovered from Preston and Campy-Line media. Also, bovine strains of *C. lanienae* in Canada typically cannot be isolated on CCDA, the most common medium used in hospitals to isolate campylobacter from human stool samples (Inglis *et al.*, 2004). As our hospital laboratory uses Karmali agar as the routine media for the detection of *Campylobacter* in human faeces, we were able to detect the presence of *C. lanienae* in our patient.

In 2009, nearly 20 % of the 2025 *Campylobacter* isolates notified in Quebec were not identified to the species level and were reported as *Campylobacter* sp. (Bureau de surveillance et vigie, 2010), possibly due to the inability (or the lack of interest or resources) of the clinical laboratory to perform phenotypic identification tests for species of *Campylobacter* other than *C. jejuni*. On the whole it seems very likely that *C. lanienae* was the causative agent. Furthermore, given that *C. lanienae* would not typically be detected in human diagnostic facilities, despite its prevalence in cattle and pig faeces, the real burden of *C. lanienae* in humans might be underestimated and should be further investigated as a potential cause of human diarrhoea disease.

## References

[R2] Acik M. N. Karahan M. Ongor H. Cetinkaya B.(2013). Investigation of virulence and cytolethal distending toxin genes in *Campylobacter* spp. isolated from sheep in Turkey. Foodborne Pathog Dis 10 589–594. 10.1089/fpd.2012.1447 23611104

[R3] Allos B. M.(2001). *Campylobacter jejuni* infections: update on emerging issues and trends. Clin Infect Dis 32 1201–1206. 10.1086/319760 11283810

[R4] Bekal S. Gaudreau C. Laurence R. A. Simoneau E. Raynal L.(2006). *Streptococcus pseudoporcinus* sp. nov., a novel species isolated from the genitourinary tract of women. J Clin Microbiol 44 2584–2586. 10.1128/JCM.02707-05 16825387 PMC1489492

[R23] Bureau de surveillance et vigie(2010). Nombre (n) de cas déclarés et taux d'incidence (Ti) annuelle par 100,000 habitants de campylobactériose selon l'espèce, province de Québec, 2000-2009. Ministère de la santé et des services sociaux du Québec.

[R5] Carbonero A. Paniagua J. Torralbo A. Arenas-Montes A. Borge C. García-Bocanegra I.(2014). *Campylobacter* infection in wild artiodactyl species from southern Spain: occurrence, risk factors and antimicrobial susceptibility. Comp Immunol Microbiol Infect Dis 37 115–121. 10.1016/j.cimid.2014.01.001 24462184

[R6] Guévremont E. Normand V. Lamoureux L. Côté C.(2008). Genetic detection of *Campylobacter lanienae* in fecal matter and stored manure from swine and dairy cattle. Foodborne Pathog Dis 5 361–364. 10.1089/fpd.2007.0054 18767980

[R8] Hill J. E. Paccagnella A. Law K. Melito P. L. Woodward D. L. Price L. Leung A. H. Ng L. K. Hemmingsen S. M. Goh S. H.(2006). Identification of *Campylobacter* spp. and discrimination from *Helicobacter* and *Arcobacter* spp. by direct sequencing of PCR-amplified cpn60 sequences and comparison to cpnDB, a chaperonin reference sequence database. J Med Microbiol 55 393–399. 10.1099/jmm.0.46282-0 16533986

[R7] Hill J. E. Penny S. L. Crowell K. G. Goh S. H. Hemmingsen S. M.(2004). cpnDB: a chaperonin sequence database. Genome Res 14 1669–1675. 10.1101/gr.2649204 15289485 PMC509277

[R9] Inglis G. D. Kalischuk L. D.(2003). Use of PCR for direct detection of *Campylobacter* species in bovine feces. Appl Environ Microbiol 69 3435–3447. 10.1128/AEM.69.6.3435-3447.2003 12788747 PMC161499

[R11] Inglis G. D. Kalischuk L. D.(2004). Direct quantification of Campylobacter jejuni and Campylobacter lanienae in feces of cattle by real-time quantitative PCR. Appl Environ Microbiol 70 2296–2306. 10.1128/AEM.70.4.2296-2306.2004 15066825 PMC383034

[R10] Inglis G. D. Kalischuk L. D. Busz H. W.(2003). A survey of *Campylobacter* species shed in faeces of beef cattle using polymerase chain reaction. Can J Microbiol 49 655–661. 10.1139/w03-087 14735214

[R12] Inglis G. D. Kalischuk L. D. Busz H. W.(2004). Chronic shedding of Campylobacter species in beef cattle. J Appl Microbiol 97 410–420. 10.1111/j.1365-2672.2004.02313.x 15239709

[R13] Inglis G. D. Kalischuk L. D. Busz H. W. Kastelic J. P.(2005). Colonization of cattle intestines by *Campylobacter jejuni* and *Campylobacter lanienae*. Appl Environ Microbiol 71 5145–5153. 10.1128/AEM.71.9.5145-5153.2005 16151098 PMC1214653

[R14] Jay-Russell M. T. Bates A. Harden L. Miller W. G. Mandrell R. E.(2012). Isolation of *Campylobacter* from feral swine (*Sus scrofa*) on the ranch associated with the 2006 *Escherichia coli* O157:H7 spinach outbreak investigation in California. Zoonoses Public Health 59 314–319. 10.1111/j.1863-2378.2012.01465.x 22405465

[R15] Logan J. M. Burnens A. Linton D. Lawson A. J. Stanley J.(2000). *Campylobacter lanienae* sp. nov., a new species isolated from workers in an abattoir. Int J Syst Evol Microbiol 50 865–872. 10.1099/00207713-50-2-865 10758898

[R16] Navarro-Gonzalez N. Ugarte-Ruiz M. Porrero M. C. Zamora L. Mentaberre G. Serrano E. Mateos A. Lavín S. Domínguez L.(2014). *Campylobacter* shared between free-ranging cattle and sympatric wild ungulates in a natural environment (NE Spain). Ecohealth 11 333–342. 10.1007/s10393-014-0921-3 24595731

[R17] Oporto B. Hurtado A.(2011). Emerging thermotolerant *Campylobacter* species in healthy ruminants and swine. Foodborne Pathog Dis 8 807–813. 10.1089/fpd.2010.0803 21438765

[R18] Sasaki Y. Fujisawa T. Ogikubo K. Ohzono T. Ishihara K. Takahashi T.(2003). Characterization of *Campylobacter lanienae* from pig feces. J Vet Med Sci 65 129–131. 12576719 10.1292/jvms.65.129

[R19] Sasaki Y. Goshima T. Mori T. Murakami M. Haruna M. Ito K. Yamada Y.(2013). Prevalence and antimicrobial susceptibility of foodborne bacteria in wild boars (*Sus scrofa*) and wild deer (*Cervus nippon*) in Japan. Foodborne Pathog Dis 10 985–991. 10.1089/fpd.2013.1548 24161070

[R20] Schweitzer N. Damjanova I Kaszanyitzky E. Ursu K. Samu P. Tóth A. G. Varga J. Dán A.(2011). Molecular characterization of *Campylobacter lanienae* strains isolated from food-producing animals. Foodborne Pathog Dis 8 615–621. 10.1089/fpd.2010.0754 21235407

[R21] Shin E. Lee Y.(2009). Comparison of three different methods for *Campylobacter* isolation from porcine intestines. J Microbiol Biotechnol 19 647–650. 19652510

[R22] Turowski E. E. Shen Z. Ducore R. M. Parry N. M. Kirega A. Dewhirst F. E. Fox J. G.(2014). Isolation of a *Campylobacter lanienae*-like bacterium from laboratory chinchillas (*Chinchilla laniger*). Zoonoses Public Health 61 571–580. 10.1111/zph.12107 24628887 PMC4163145

